# Supplementary material for: Altered corticolimbic connectivity reveals sex-specific adolescent outcomes in a rat model of early life adversity
Source: eLife. 2020 Jan 20;9:e52651. doi: 10.7554/eLife.52651 (PMC7010412; doi:10.7554/eLife.52651)
Supplement: Supplementary file 1. [file elife-52651-supp1.docx]

**Supplementary File 1**: Table of non-significant results illustrated in Figure 2

| **Measure** | **Source** | **df** | **F** | **p** | **partial η2** |
| --- | --- | --- | --- | --- | --- |
| **Male**  **PL5**  **Innervation** | Age | 2 | 1.097 | 0.343 | 0.051 |
|  | Rearing | 1 | 0.375 | 0.544 | 0.009 |
|  | Age x Rearing | 2 | 1.695 | 0.196 | 0.076 |
|  | Error | 41 |  |  |  |
| **Male**  **PL2**  **Innervation** | Age | 2 | 1.665 | 0.202 | 0.075 |
|  | Rearing | 1 | 4.365 | 0.043 | 0.096 |
|  | Age x Rearing | 2 | 3.236 | 0.05 | 0.136 |
|  | Error | 41 |  |  |  |
| **IL**  **Innervation** | Sex | 1 | 0.567 | 0.454 | 0.007 |
|  | Age | 2 | 2.111 | 0.128 | 0.051 |
|  | Rearing | 1 | 3.321 | 0.072 | 0.040 |
|  | Age x Rearing | 2 | 1.353 | 0.264 | 0.033 |
|  | Sex x Rearing | 1 | 1.007 | 0.319 | 0.013 |
|  | Age x Sex x Rearing | 2 | 1.922 | 0.153 | 0.046 |
|  | Error | 79 |  |  |  |
| **Female**  **IL**  **Innervation** | Age | 2 | 0.179 | 0.837 | 0.009 |
|  | Rearing | 1 | 3.627 | 0.064 | 0.087 |
|  | Age x Rearing | 2 | 2.213 | 0.123 | 0.104 |
|  | Error | 39 |  |  |  |
| **IL5**  **Innervation** | Sex | 1 | 1.379 | 0.244 | 0.017 |
|  | Age | 2 | 1.913 | 0.154 | 0.046 |
|  | Rearing | 1 | 1.740 | 0.191 | 0.022 |
|  | Age x Rearing | 2 | 1.778 | 0.176 | 0.043 |
|  | Sex x Rearing | 1 | 1.460 | 0.230 | 0.018 |
|  | Age x Sex x Rearing | 2 | 1.756 | 0.179 | 0.043 |
|  | Error | 79 |  |  |  |
