# Supplementary material for: Altered corticolimbic connectivity reveals sex-specific adolescent outcomes in a rat model of early life adversity
Source: eLife. 2020 Jan 20;9:e52651. doi: 10.7554/eLife.52651 (PMC7010412; doi:10.7554/eLife.52651)
Supplement: Supplementary file 2. [file elife-52651-supp2.docx]

**Supplementary File 2**: Table of non-significant results illustrated in Figure 3

| **Measure** | **Source** | **df** | **F** | **p** | **partial η2** |
| --- | --- | --- | --- | --- | --- |
| **Male**  **Seconds in Open Arm (EPM)** | Age | 2 | 1.691 | 0.189 | 0.031 |
|  | Rearing | 1 | 0.204 | 0.652 | 0.002 |
|  | Age x Rearing | 2 | 1.533 | 0.221 | 0.028 |
